# Novel Characterization of Myeloid-Derived Suppressor Cells in Tumor Microenvironment

**DOI:** 10.3389/fcell.2021.698532

**Published:** 2021-08-30

**Authors:** Yanan Li, Hongdan He, Ribu Jihu, Junfu Zhou, Rui Zeng, Hengxiu Yan

**Affiliations:** ^1^Immunotherapy Laboratory, College of Pharmacology, Southwest Minzu University, Chengdu, China; ^2^Immunotherapy Laboratory, Qinghai Tibet Plateau Research Institute, Southwest Minzu University, Chengdu, China

**Keywords:** tumor microenvironment, myeloid-derived suppressor cells, targeted therapy, multivariate effects, regulation

## Abstract

Myeloid-derived suppressor cells (MDSCs) are a heterogeneous group of cells generated in various pathologic conditions, which have been known to be key components of the tumor microenvironment (TME) involving in tumor immune tolerance. So MDSCs have been extensively researched recently. As its name suggests, immunosuppression is the widely accepted function of MDSCs. Aside from suppressing antitumor immune responses, MDSCs in the TME also stimulate tumor angiogenesis and metastasis, thereby promoting tumor growth and development. Therefore, altering the recruitment, expansion, activation, and immunosuppression of MDSCs could partially restore antitumor immunity. So, this view focused on the favorable TME conditions that promote the immunosuppressive effects of MDSCs and contribute to targeted therapies with increased precision for MDSCs.

## Introduction

The tumor microenvironment (TME) is the direct environment in which tumor cells live, consists of lymphocytes, immune cells, stromal cells, and extracellular matrix (ECM), and it is closely associated with tumor growth, invasion, and metastasis (Chen et al., 2015). A series of tumor-promoting cells exist in the TME, including T regulatory cells (Tregs), T helper type 2 cells, tumor-associated macrophages (TAMs), and myeloid-derived suppressor cells (MDSCs). The cytokines and chemokines secreted by these cells create an immunosuppressive circumstance that prevents immune cells from functioning. Therefore, TME provides a permissive environment for the progression and metastatic dissemination of tumor cells (Ugel et al., 2015). And, MDSCs are currently considered to be major players in the development of tumor immune tolerance. At present, studies have shown that cytokines from tumor cells and activated immune cells in the TME promote the recruitment, activation, expansion, and suppressive activities of MDSCs in tumor progression (Table 1). These cytokines are divided into two groups in light of the different roles on MDSCs. The first class is in charge of the expansion of MDSCs, and it mainly includes vascular endothelial growth factor (VEGF), granulocyte-macrophage colony-stimulating factor (GM-CSF), macrophage colony-stimulating factor (M-CSF), and granulocyte colony-stimulating factor (G-CSF). The second class plays a remarkable part in the MDSC activation procedure and mainly includes interferon-γ (IFN-γ), high-mobility group box 1 (HMGB1), tumor necrosis factor (TNF), and interleukin-1β (IL-1β), IL-4, IL-6, and IL-13 (Umansky and Sevko, 2013; Condamine et al., 2015). In addition, there are some newly discovered factors, such as endoplasmic reticulum (ER) stress and tumor-derived exosomes (TEXs), which are also implicated as key factors that regulate MDSCs to play a tumor-promoting aspect in the TME. Concurrently, MDSCs in the TME directly enhance tumor angiogenesis and migration in addition to facilitating immune response (Table 1). MDSCs have become one of the main impediments to effective cancer immunotherapy and have been considered as valuable markers of predicting cancer progression in numerous clinical studies. Therefore, extensive efforts in the development of targeting MDSC therapies are ongoing vigorously (Betsch et al., 2018; Li et al., 2018).

**TABLE 1:** Myeloid-derived suppressor cells in a variety of tumors.

Tumor	Contribution of MDSCs to tumor development	Regulatory effect of factors on MDSC	Reference
Prostate cancer	MDSCs promoted tumor survival	Tumor-derived G-CSF promoted the proliferation of MDSCs via a STAT3-dependent pathway	Yu et al., 2015
	MDSCs promoted tumor progression	Chemokines promoted the expansion of CCR5^+^PMN-MDSCs at the BM, and potentiated their immunosuppression at the tumor site	Hawila et al., 2017
	MDSCs promoted tumor angiogenesis	CSF1R signaling promoted tumor recruitment of M-MDSC recruitment from peripheral blood	Priceman et al., 2010
Breast cancer	MDSCs promoted tumor growth	TEXs with abundant PGE2 and TGF-β enhanced the expansion and immunosuppression of MDSCs	Xiang et al., 2009
	MDSCs promoted tumor progression	Transmembrane Tm-TNF-α induced the immunosuppression of MDSCs	Hu et al., 2014
	MDSCs promoted tumor growth and metastasis	Mir-494 induced the expansion of MDSCs in tumor tissues by increasing the activity of the Akt pathway	Liu et al., 2012
	MDSCs stimulated tumor cell metastasis to distant sites	IL-6 secreted from breast cancer cells facilitated MDSC recruitment	Oh et al., 2013
Melanoma	MDSCs promoted tumor growth	Lnc-chop encouraged the activity of C/EBP β, improved the immunosuppression of MDSCs	Gao et al., 2018
	MDSCs promoted tumor progression	Tumor-derived chemokines CCL3, CCL4, and CCL5 recruited CCR5^+^ MDSCs to the tumor site	Blattner et al., 2018
	PMN-MDSCs induced the proliferation and EMT of tumor cells	Tumor-derived chemokines CXCL1, CXCL2, and CXCL5 recruited CXCR2^+^ MDSC to the tumor site	Toh et al., 2011
Colon cancer	MDSCs promoted tumor growth	HMGB1 promoted the differentiation of MDSCs from bone marrow progenitor cells, and activated the immunosuppression of MDSCs via the NF-κB pathway	Parker et al., 2014
	MDSCs promoted tumor growth	MiR-200c promoted immunosuppression of MDSCs by targeting PTEN/FOG2, which led to STAT3 and PI3K/Akt activation	Mei et al., 2015
	MDSCs formed PMN in the pre-metastatic liver	VEGFA secreted by colon cancer cells stimulated CXCL1 production by TAMs, which recruited CXCR2^+^ MDSCs to promote liver metastasis.	Wang et al., 2017

As mentioned above, there are many factors existed in the TME for MDSC recruitment, activation, and expansion, which may be targets to the cancer treatment by modifying MDSC function. This review highlighted the recruitment, expansion, and activation of MDSC in the TME and may provide more effective strategies for MDSC-based cancer therapy.

## Definition of MDSCs

Myeloid-derived suppressor cells are derived from myeloid progenitors and immature myeloid cells (IMCs). Under physiological conditions, they rapidly differentiate into mature granulocytes, dendritic cells (DCs), and macrophages, then migrate the corresponding peripheral organs and tissues from the bone marrow to exert normal immune functions. Nevertheless, under pathological situations, such as cancer, infection, inflammation, sepsis, and surgical injury, the maturation of these myeloid-derived progenitors is blocked by cytokines, so they stay in various differentiation stages to become MDSCs with immunosuppressive function, which are also recruited, migrated and amplified under the action of cytokines, throughout the whole process of disease occurrence (Gabrilovich and Nagaraj, 2009). Of note, MDSC expansion does not exclusively result from myelopoiesis in the bone marrow, but also the differentiation of MDSC progenitors as well as reprogramming of monocytes and neutrophils in peripheral tissues (Bergenfelz et al., 2015; Heine et al., 2017; Yaseen et al., 2021). In 1995, CD11b^+^/Gr-1^+^ myeloid cells were found to be involved in tumor immune escape and development, and they were described as MDSCs in 2007 (Gabrilovich et al., 2007). Traditionally, two subpopulations of MDSCs are shown to exist, namely, granulocytic CD11b^+^Ly6G^+^Ly6C^*lo*^ [G-MDSCs or polymorphonuclear (PMN)-MDSCs] and monocytic CD11b^+^Ly6G^–^Ly6C^*hi*^ (M-MDSCs), in mouse (Bronte et al., 2016). In human, M-MDSCs are characterized as CD11b^+^CD33^+^CD14^+^HLA-DR^*lo/*–^CD15^–^ and PMN-MDSCs as CD11b^+^CD33^+^CD15^+^CD66b^+^HLA-DR^*lo/*–^ (Bronte et al., 2016; Elliott et al., 2017). Besides, a group of IMCs was found in human peripheral blood which was referred to as early stage MDSCs. Lin cocktail, including CD3, CD14, CD15, CD19, and CD56, could be used to differentiate early stage MDSCs from MDSCs, and early stage MDSCs are characterized as Lin^–^HLA-DR^–^CD33^+^ in human (Almand et al., 2001; Bronte et al., 2016). Another novel subpopulation of tumor-induced MDSCs was identified in the peripheral blood of a patient with metastatic pediatric sarcoma, which shares the fibrocytes phenotypic and functional characteristics (Zhang et al., 2013). And, Zoso et al. (2014) found this subpopulation simultaneously expressing surface markers of MDSCs, DCs as well as fibrocytes, which were defined as fibrocystic MDSCs (CD11b^*low*^CD11c^*low*^CD33^+^IL-4Ra^+^). Recently, a novel group of MDSCs with immature eosinophilic phenotype was found to accumulate at the site of infection to exacerbate the chronic Staphylococcus infection in mice, which defined as eosinophilic MDSCs (Eo-MDSCs) by Goldmann et al. (2017), characterizing as SSC^*high*^ Ly6C^*low*^ Ly6G^–^CCR3^*low*^ Siglec-F^*low*^ IL-5R^*low*^. These new MDSCs subpopulations enrich the diversity of MDSCs, which attract researchers to study and classify MDSCs more carefully to promote the development of targeting MDSCs treatment.

With the ongoing advance in research on MDSCs, several other potential markers have been identified. For example, CD84 and CD36 have been used to identify MDSCs, while CD244, fatty acid transport protein 2 (FATP2) are thought to more effectively distinguish M-MDSCs from PMN MDSCs in mice (Al-Khami et al., 2017; Veglia et al., 2019, 2021; Alshetaiwi et al., 2020). And, in human, CD84 and S100A9 are also suggested to identify MDSCs, CD66b is used to distinguish PMN-MDSCs from M-MDSCs. Of note, lectin-type oxidized LDL receptor 1 (LOX-1), as a specific marker for human PMN-MDSCs, is used to distinguish PMN-MDSCs from M-MDSCs and normal neutrophils (Zhao F. et al., 2012; Condamine et al., 2016; Lin et al., 2018). In a recent study, by using single-cell RNA-seq (scRNA-seq), Alshetaiwi et al. (2020) found that there were 642 differentially expressed genes between PMN-MDSCs and normal neutrophils as well as 223 differentially expressed genes between M-MDSCs and normal monocytes in the MMTV-PyMT mouse breast cancer model, revealing that MDSCs were quite different from normal myeloid cells. At the same time, there was a large overlap between the genomes of PMN-MDSCs and M-MDSCs involved in immunosuppression, such as IL-1B, ARG-2, CD84, and WFDC17, and chemokine receptors, such as CCR2 and CXCR2, revealing that MDSCs could be migrated to the primary tumor by tumor-derived chemokines. Of note, CD84 could be identified as a specific surface marker of MDSCs in breast cancer, but whether it can be used to identify MDSCs in other cancers needs further test (Alshetaiwi et al., 2020; Veglia et al., 2021). In the future, bulk or single-cell genomics could be considered to accurately identify MDSCs cell surface markers and specific genomic features in different types of malignancies, which could help identify potential therapeutic targets and improve cancer treatment by targeting MDSCs.

## Contribution of MDSCs To Tumor Development

### MDSC Immunosuppression

**FIGURE 1:**
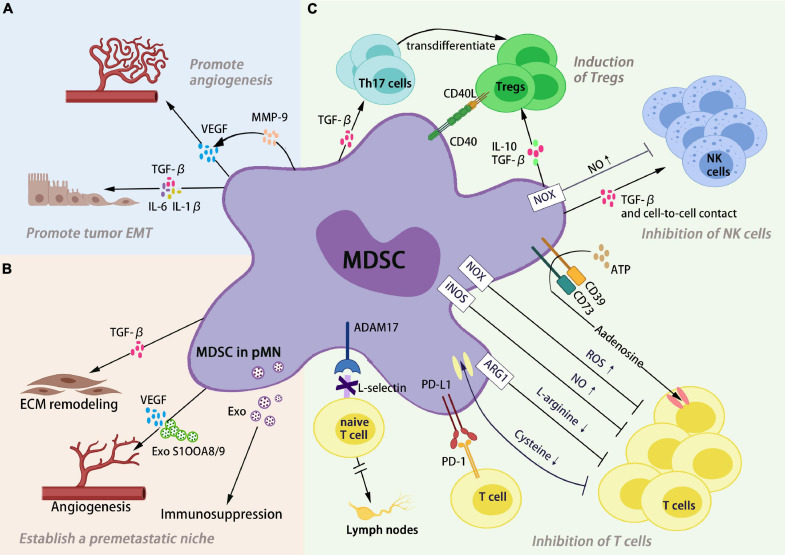
Myeloid-derived suppressor cells (MDSCs) promote tumor development through different pathways. **(A)** MDSC non-immunologic functions. MDSCs in the TME promote tumor progression by promoting angiogenesis and EMT of tumors. **(B)** MDSC establish a pre-metastatic niche. MDSC-derived factors participate in the stepwise establishment of the PMN, resulting in a “soil” that supports the colonization of CTCs. **(C)** MDSC immunosuppression. MDSCs inhibit antitumor immunity by inhibiting T cells and natural killer cell proliferation and function and inducing Treg recruitment.

As important immunosuppressive cells in the TME, MDSCs inhibit antitumor immunity by inhibiting T cells and natural killer cells proliferation and function and inducing Treg recruitment. Thus, the tumor cells escape the immune surveillance and in turn promote the development of tumors (Figure 1).

Myeloid-derived suppressor cells exert immunosuppressive effects by depleting the fundamental amino acids, including L-arginine and cysteine, that are cardinal for T cell function in the TME. MDSCs have a high expression of arginase-1 (ARG-1) and inducible NO synthase (iNOS) in the TME. Decomposition of L-arginine by iNOS produces large amounts of NO and L-citrulline, while ARG-1 converts L-arginine to L-ornithine (Consonni et al., 2019). Depletion of L-arginine and generation of large amounts of NO in the TME leads to downregulated expression of the TCR complex CD3 ζ chain and arrest of T-cell proliferation (Yang et al., 2020). T cells depend on macrophages and DCs to take up cysteine from the extracellular medium. Under normal circumstances, antigen presenting cells take up extracellular oxidized cysteine, which is converted into cysteines and then presented to T cells that provide conditions for the activation versus proliferation of T cells. By taking up cysteine, MDSCs reduce cysteine levels in the TME, leading to impaired T-cell activation (Srivastava et al., 2010). MDSCs drive reactive oxygen species (ROS) production by upregulating NADPH oxidase activity, particularly NOX2 subunit 47 (phox) and gp91 (phox). The immunosuppressive effect of ROS on T-cell function has been widely demonstrated, and studies have found that the administration of ROS inhibitors counteracted the suppressive effect of human MDSCs on T cells (Corzo et al., 2009; Wei et al., 2015). Besides, ROS by MDSCs relying on NOX2 supports MDSC expansion and recruitment in the TME by upregulating VEGF receptors on MDSCs to further promote tumor development (Kusmartsev et al., 2008; Corzo et al., 2009). By contrast, MDSCs prohibit the homing of naive CD4^+^ and CD8^+^ T cells to lymph nodes, which in turn interfere with T-cell activation. The underlying mechanism is that MDSCs express the adisintegrinandmetalloproteinase17 (ADAM17) to downregulate the L-selectin level on the membrane surface of CD4^+^ and CD8^+^ T cells (Hanson et al., 2009). MDSCs were found in multiple tumor models of mice and in patients with cancer to have been able to increase programmed cell death ligand 1 (PD-L1) expression, promote T-cell anergy by interacting with the programmed cell death protein 1 (PD-1) on T cells infiltrating tumor lesions, and in turn to drastically downregulate T cell-mediated antitumor reactivity (Weber et al., 2018). Fuse et al. (2016) reported that PD-L1 blockade decreased the immune suppression ability of MDSCs on T cells. Moreover, recent studies have found that MDSCs mediate their suppression on T cells through adenosine. MDSCs from tumor tissues of patients with cancer increased adenosine production through considerable upregulation of CD39 and CD73 *in vitro*, and adenosine signals mainly through A2A type and A3 type adenosine receptors to suppress T cell activation and immune response (Umansky et al., 2014; Li et al., 2018).

In addition to inhibiting T cells, MDSCs inhibit NK cell cytotoxicity as a tumor immune evasion mechanism. Overexpression of indoleamine 2,3-dioxygenase (IDO) by MDSCs reduces tryptophan levels in the TME, and this phenomenon not only stimulates the differentiation of Tregs from naive T cells but also induces NK cell apoptosis (Fleming et al., 2018). MDSCs suppress NK cell cytotoxicity by expressing the immunosuppressive cytokine TGF-β, including the suppression of NKG2D expression and IFN-γ secretion of NK cells (Trikha and Carson, 2014). MDSCs were recently found in the co-culture of autologous NK cells and MDSCs from patients with cancer to inhibit NK cell downstream effector functions, including cytokine production and antitumor activity, which was dependent on NO produced by MDSCs (Stiff et al., 2018).

T regulatory cells are a group of T-cell subsets downregulating immune responses; they promote the immune escape of tumors primarily by releasing inhibitory cytokines or by cell-to-cell contact. MDSCs produce the CCR5 ligands CCL4 and CCL5, which recruit Tregs to tumor tissues by CCR5 receptors highly expressed on Treg surface (Qu et al., 2016). Schlecker et al. (2012) have shown that the intratumoral injection of CCL4 or CCL5 increased the number of Tregs in the TME, whereas the lack of CCR5 resulted in a substantial reduction in Treg recruitment to the tumor. MDSCs in the TME overexpress IDO, an important enzyme-degrading tryptophan, which induces naive T cells to differentiate toward Treg phenotype by reducing tryptophan levels (Fallarino et al., 2006). Huang et al. (2006) demonstrated that IFN-γ secreted by T cells stimulated MDSCs in tumor to enhance their IL-10 and TGF-β secretion levels, which induce the activation of Tregs dependent on IL-10 and IFN-γ secreted by MDSCs. Besides, the interaction between CD40 and CD40L is critical for the induction of Tregs. Early studies have shown that MDSCs are recruited Tregs by expressing CD40 and the lack of CD40 results in significantly reduced Treg expansion (Pan et al., 2010). Apart from MDSCs inducing the development of Tregs, Th17 cells are induced by MDSCs to transdifferentiate into Tregs, which is dependent on human MDSC-derived TGF-β and retinoic acid (Hoechst et al., 2011).

### MDSC Non-immunologic Functions

Besides modulating the immune system through immunosuppression, the MDSCs in the TME could contribute to the progression of the primary tumor through non-immunological functions, including promoting angiogenesis and metastasis of tumor (Figure 1). As shown in the mouse model, the co-injection of Gr-1^+^CD11b^+^ cells and tumor cells stimulated tumor angiogenesis (Yang et al., 2004). VEGF is a potent angiogenic factor that promotes tumor angiogenesis. Kujawski et al. (2008) have found that MDSCs from mouse tumors increased VEGF production through the activation of signal transducer and the activator of transcription 3 (STAT3) (Li et al., 2019). In another study, MDSCs also increased VEGF levels by expressing matrix metalloproteinase-9 (MMP-9), which further improves angiogenesis (Yang et al., 2004). In addition to promoting tumor angiogenesis, MDSCs also promote tumor cells to acquire a mesenchymal phenotype by secreting inflammatory factors Hepatocyte Growth Factor (HGF), TGF-β, IL-6, and IL1-β, which lead to the reduction or deletion of E-cadherin in tumor cells, and drive tumor cell epithelial mesenchymal transition (EMT) (Toh et al., 2011; Ouzounova et al., 2017; Pastaki Khoshbin et al., 2019). Additionally, MDSCs play an indispensable role in establishing the pre-metastatic niche (pMN). Primary tumors release signals before metastasis to regulate secondary organ resident cells or recruitment cells, including neutrophils, macrophages, and MDSCs. These cells respond to signals that transform healthy secondary organs and tissues into a “soil” that supports the colonization of circulating tumor cells (CTCs) (Peinado et al., 2017; Nasrollahzadeh et al., 2020; Wu et al., 2020). VEGF^+^ MDSCs at tumor specific pre-metastatic sites provided a favorable microenvironment for the entry of CTCs, which was first identified by Kaplan et al. (2005). And, Yan et al. (2010) found MDSCs in the lungs of breast cancer model mice increased significantly 2 weeks before CTCs arrival and was associated with decreased lung immune function. The liver, like the lung, is a metastatic target organ for major malignancies. Infiltration of MDSCs in pre-metastatic liver tissue has been found in a variety of tumor models such as pancreatic and colorectal cancer (Kruger, 2015). Further studies revealed that MDSCs-derived exosomes, TGF-β, S100A8/A9, and VEGF induce angiogenesis, ECM remodeling, and immunosuppression to promote PMN formation and metastasis (Hsu et al., 2019; Wang et al., 2019). Metastasis has become a leading cause of cancer-related death. So, targeting MDSCs treatments hold promise to halt PMN formation and progression, prolonging the survival of tumor patients.

Taken together, the MDSCs in the TME contribute to the development of tumors and immune escape through multiple pathways, which are generally related to poor patient prognosis. MDSC-based cancer therapy has thus become a major research direction to reestablishing anticancer immunity, and it is thriving.

## MDSC Recruitment, Expansion, and Activation in TME

### Factors Affecting MDSC Recruitment

Chemokines are a class of small secreted proteins that regulate the migration of immune cells, and more than 50 human chemokines have been currently discovered since they were first named chemokines in 1986 (Schulz et al., 2016). In accordance with the differences in the sequence of the first two conserved cysteines at the N-terminus of their molecules, chemokines are classified into four subfamilies: CXC, CC, C, and CX3C (Li et al., 2020). Several studies have demonstrated that chemokines expressed by cancer cells induce MDSC recruitment into the TME by binding to their specific receptors on MDSCs.

CCL2 is a necessary member of the CC family of chemokines, also described as monocyte chemotactic protein-1 (MCP-1), with a high affinity to the chemokine receptor CCR2 found on the MDSC cell membrane (Behfar et al., 2018). In a study of a mouse glioma model, M-MDSCs were found to be recruited to the tumor site by CCL2 that produced by microglia and macrophages in the TME, whereas MDSC infiltration into the tumor was significantly reduced in CCL2-deficient mice (Chang et al., 2016). Human breast, ovarian, and gastric tumor cells cultured *in vitro* secrete CCL2; the cognate MDSCs from these patients express CCR2; and the recruitment of MDSCs to the tumor site is mediated by CCL2-CCR2 signaling. Similarly, MDSC recruitment to tumor tissues via CCL2-CCR2 signaling has been found in hepatocellular carcinoma (HCC) (Huang et al., 2007; Lesokhin et al., 2012). In a mouse model of intraosseous prostate cancer, CCL2 signaling was blocked using neutralizing anti-CCL2 antibodies alone or in combination with the chemotherapeutic drug docetaxel; the results indicated that CCL2 blockade inhibits prostate cancer development and the effect is more profound when combined with docetaxel (Kirk et al., 2013). Thus, the critical role of CCL2-CCR2 signaling in MDSC recruitment and tumor progression makes it a promising target for anticancer therapy.

CCR5 is a cell membrane protein whose ligands include CCL3, CCL4, and CCL5. In a melanoma mouse model and patients with melanoma, the CCR5^+^ MDSCs accumulated in tumor tissues were positively correlated with the upregulation of CCL3, CCL4, and CCL5. These CCR5^+^ MDSCs exhibited stronger suppressive activity during the progression of tumors (Blattner et al., 2018). Similarly, in TRAMP-C1 prostate tumors, CCR5 ligands induced the expansion of MDSCs in the bone marrow, and CCR5 drove the migration of MDSCs from the bone marrow into the blood and finally their recruitment at the tumor site (Hawila et al., 2017). CCR5 blockade decreased the percentage of MDSCs and inhibited melanoma proliferation, mainly through upregulating suppressor of cytokine signaling 3 (SOCS3) expression, which in turn inhibited the IL-STAT3 pathway (Hawila et al., 2017). In addition, chemokine receptors are more effective targets than chemokines, and targeting chemokine receptors is a promising therapeutic means because multiple chemokines generally correspond to a single chemokine receptor, such as CCL2/CCL12-CCR2, CCL3/4/5-CCR5, and CXCL5/CXCL2/CXCL1-CXCR2 (Bronte et al., 2016).

CXCR2 was cloned from a human neutrophil cell line in 1991, and it is also known as interleukin-8 receptor B (IL8RB) because of its ability to bind non-specifically to IL-8 (Murphy and Tiffany, 1991). The CXCR2 expressed by MDSCs has three chemokine ligands in the TME, including CXCL5, CXCL2, and CXCL1. In bladder cancer, tumor cells secreted CXCL2-stimulating nuclear factor kappa B (NF-κB) pathways in MDSCs to induce MDSC accumulation in the TME via CXCL2-CXCR2 signaling (Zhang et al., 2017). A study by Wang et al. (2017) on liver metastasis of colorectal cancer in mice has suggested that CXCL1-CXCR2 promotes tumor liver metastasis. Mechanistically, colorectal cancer cells stimulate TAMs to produce CXCL1 by secreting VEGFA, and CXCL1 recruits CXCR2^+^ MDSCs from the blood into the pre-metastatic liver. In a prostate adenocarcinoma model, heterotypic CXCL5-CXCR2 signaling upregulated and activated YAP1, consequently recruiting MDSCs into tumor tissues. Further study found that blocking CCR2 inhibits tumor development (Wang et al., 2016).

### Major Signaling Pathways Associated With MDSC Expansion and Activation in TME

The expansion and activation of MDSCs in the TME involve multiple signaling pathways, among which AMP-activated protein kinase (AMPK) is a potential regulator of MDSC functions. AMPK is mainly responsible for regulating energy metabolism as well as immune system regulation, and its expression is downregulated during the progression of tumor development (Pineda et al., 2015). AMPK activation regulates downstream immune signaling pathways, thereby affecting the function of immune cells. A study by Trikha et al. (2016) found that the use of AMPK activators was able to reduce the levels of MDSCs in the spleen and tumors. In addition, studies have shown that AMPK activation inhibits its downstream signaling pathways NF-κB and STAT signaling pathways, while NF-κB and STAT signal pathways are essential for the expansion and activation of MDSCs in the TME (Salminen et al., 2011; Rutherford et al., 2016).

The expansion and activation of MDSCs in the TME are mainly induced by cytokines secreted by tumor cells or activated immune cells, such as VEGF, GM-CSF, M-CSF, G-CSF, IL-1β, IL-4, IL-6, and IFN-γ. STAT1, -3, -5, and -6 play a distinct role in the MDSC immunosuppression induced by the above cytokines. STATs belong to a family of transcription factors with dual functions of signal transduction and transcription. Upon stimulation with M-CSF, IL-6, GM-CSF, and VEGF by tumor cells, STAT signaling regulates Tregs, TAMs, and MDSCs, consequently exerting a tumor-promoting effect (Ko and Kim, 2016).

Signal transducer and the activator of transcription 3 is implicated as a major driver promoting MDSC expansion, and multiple cytokines in the TME all promote MDSC proliferation and survival by activating STAT3 (Gabrilovich et al., 2012). Colony-stimulating factors are essential in the regulation of myeloid cell differentiation. STAT3 was described to improve the expansion of intratumoral MDSCs in conjunction with other factors, such as GM-CSF, M-CSF, and G-CSF. In addition, the G-CSF secreted by tumor cells induced MDSC recruitment and decreased their generation number by using STAT3 inhibitor. Further study by Yu et al. (2015) found that SOCS3 attenuated the effect of G-CSF on MDSC recruitment by blocking the induction of STAT3 activation. In another study, GM-CSF and G-CSF activated STAT3 to induce the downregulation of IFN-related factor-8 (IRF-8). As a transcription factor, IRF-8 not only induces monocyte and DC development but also restricts granulocyte development. Thus, inhibition of IRF-8 is associated with a block in MDSC differentiation and an increased number of MDSC (Waight et al., 2013). Furthermore, tumor releases GM-CSF and IL-6 promotes the conversion of myeloid cells to an MDSC phenotype, mainly through the activation of a CCAA T-enhancer-binding protein β(C/EBP β)-mediated program that implicates the downstream blockade of STAT3 for terminal differentiation (Marigo et al., 2010; Zhang et al., 2010). A high secretion level of GM-CSF, which is present in a range of tumor entities, such as pancreatic cancer, has been demonstrated to stimulate the accumulation of MDSCs in the TME. Blocking GM-CSF by using neutralizing antibodies or antagonists in in-vitro tumor models also inhibited the expansion of MDSCs and their suppressive activity on T cells (Gargett et al., 2016). Interestingly, pre-clinical and clinical evidence suggested that the role of GM-CSF on MDSCs is related to its concentration level. *In vitro*, the time to generate MDSCs from mouse bone marrow cells cultivated in GM-CSF was inversely correlated with GM-CSF concentration (Lutz et al., 2000). All of the above emphasized that the GM-CSF from the TME promoted the expansion of MDSCs. The VEGF in the TME not only promotes tumor angiogenesis but also induces the activation of MDSCs. As early as Gabrilovich et al. (1996) have shown that VEGF secreted by different cancer cells could affect the functional maturation of myeloid progenitor cells, especially inhibiting the maturation of DCs. In a recent study, a murine ovarian tumor cell line overexpressing VEGF stimulated the expansion of MDSCs in the TME while reducing the number of effector T cells (Horikawa et al., 2017). A further study has suggested that VEGF induced MDSC expansion through VEGFR-2/STAT 3 signaling, and the activation of STAT3 induced VEGF expression, which in turn formed a positive feedback loop (Bartoli et al., 2003; Zhao et al., 2015). IL-6 has also been reported to be one of the important cytokines that mediate MDSC expansion via STAT3. In mice, the overexpression of peroxisome proliferator-activated receptor γ (PPARγ), which is defined as an anti-inflammatory molecule, could upregulate the IL-6 level to activate STAT3 and expand MDSCs. Further study has demonstrated that in a mouse model of breast cancer, MDSCs secreted IL-6 at the tumor site, thus inducing PSTAT3 expression by tumor cells and promoting tumor progression and metastatic potential (Oh et al., 2013). In addition, STAT3 upregulates the expression of S100A8/9, which is considered as a pro-inflammatory protein, and leads to the inhibition of DC differentiation, consequently leading to MDSC expansion. NOX2 expression is required for S100A8/A9 upregulation mediated by STAT3, while NOX2 activation also inhibits the immune response of T cells (Cheng et al., 2008; Zheng et al., 2015).

Signal transducer and the activator of transcription 6 is a downstream transcription factor for IL-4R and IL-13R, while IL-4 and IL-13 bind to IL-4R α kinase subunit and induce the activation of MDSCs (Gallina et al., 2006). Another study proved that STAT6 activation associated with IL4-Rα induces TGF-β secretion and ARG-1 expression to mediate immunosuppression (Gabrilovich and Nagaraj, 2009). Furthermore, in the STAT6^–/–^ mouse model, the MDSCs in the body exhibited diminished suppressive activity due to reduced ARG-1 expression (Munera et al., 2010).

Previous studies demonstrated that blocking the secretion of IFN-γ from T cells eliminated MDSC immunosuppression by blocking the upregulation of iNOS (Gallina et al., 2006). A further study found that MDSC activation by IFN-γ is dependent on STAT1 signaling. Mechanistically, IFN-γ activated the transcription of IRF1 by inducing STAT1 phosphorylation, consequently inducing the upregulation of PD-L1 expression on MDSCs (Lu et al., 2016).

Besides STAT-related signaling pathways, the NF-κB pathway is a remarkable factor in stimulating the activation of MDSCs, and the cytokines associated with it include TNF-α and IL-1 β. TNF-α is an inflammatory cytokine enriched in the TME, and it is related to the accumulation and suppressive activity of MDSCs. Transmembrane TNF-α (Tm-TNF-α) is the main ligand of TNFR2, and the binding between Tm-TNF-α and TNFR2 activates MDSC immunosuppression, as evidenced by upregulating ARG-1 and iNOS to promote the secretion of NO. Further study proved that the induction of MDSC immunosuppression by Tm-TNF-α was dependent on the activation of the NF-κB signaling pathway by IκBα degradation and the translocation of NF-κB p65 (Hu et al., 2014). Another study has analogously demonstrated that TNFR-2 activated NF-κB signaling, which in turn promoted MDSC survival by upregulating cellular FLICE inhibitory protein (c-FLIP) and inhibiting caspase-8 activity (Zhao X. et al., 2012). Considering IL-1 is a key downstream mediator of inflammation, it plays a leading role in the progression of tumor development. An early study has shown that the transfection of murine 4T1 breast cancer cells with the proinflammatory cytokine IL-1 β created a chronic inflammatory microenvironment at the tumor site, resulting in elevated levels of MDSCs and shortened survival of mice (Bunt et al., 2006). Similarly, Tu et al. (2008) have shown that IL-1 β was associated with gastric cancer development and mainly activated MDSCs *in vitro* and *in vivo* through the IL-1RI/NF-κB pathway, thereby inducing immunosuppression and promoting tumor development.

Toll-like receptors (TLRs) are recognized as critical factors involved in tumor pathogenesis, with a high probability of activating various signaling pathways during cancer progression. The TLR family induces NF-κB activation mainly dependent MyD88, which in turn activates immunosuppression in MDSCs. MDSCs lacking MyD88 lost their immunosuppression and even gained immunostimulatory activity in the TME (Hong et al., 2013). HMGB1 is a highly conserved nuclear protein that is released by some necrotic cells as an inflammatory mediator in the TME, and is it also a factor contributing to MDSC immunosuppression. Parker et al. (2014) have shown that HMGB1 in the TME regulated the MDSC level and immunosuppression by activating the NF-κB pathway. HMGB1 also promoted the differentiation of MDSCs by contributing to its inhibition of CD4^+^ and CD8^+^ T-cell activation.

### Effect and Mechanism of TEX on MDSCs

Tumor-derived exosomes are exosomes secreted by tumor cells, and they have attracted much attention in recent years. Exosomes are a sort of EVs that could be secreted from many different cells, such as erythrocytes, lymphocytes, and tumor cells (Whiteside, 2016). Exosomes contain nucleic acids, proteins, and lipids, and different content loadings into exosomes rely on different sorting mechanisms. As a kind of important vesicles in human body, exosomes can be associated with almost any disease. Since 2013, exosomes have gradually become a research hotspot of disease markers, disease mechanisms, and drug development. Multiple favorable conditions exist in the TME and promote TEX formation and release, including extracellular acidity, hypoxia, and genotoxic stress. TEXs have been reported to regulate the expansion and immunosuppression function of MDSCs in different tumors. For example, TEXs released by melanoma cells inhibit the ability of normal monocytes to differentiate into DCs, consequently supporting the accumulation of MDSC in the TME (Filipazzi et al., 2012). Therefore, investigating the mechanism of TEXs on MDSCs may provide a new direction to target MDSCs and control tumor development.

Prostaglandin E2 (PGE2) and TGF-β conveyed by TEXs are necessary for the amplification and activation of MDSC in tumors. This class of TEXs induces MDSC accumulation, which promotes tumor progression. Meanwhile, further study has found that blocking PGE2 and TGF-β inhibited the induction of the effect of these exosomes on MDSCs and then attenuate the tumor immune escape mediated by MDSCs (Xiang et al., 2009). Chalmin et al. (2010) found that TEXs promoted MDSC immunosuppression rather than their expansion by STAT3 activation, which was triggered by TEXs membrane-associated heat shock protein 72 in a TLR2/MyD88 dependent manner. This study also found that dimethyl amiloride promoted the antitumor effect of the chemotherapeutic drug cyclophosphamide by blocking the immunosuppression of MDSCs through depletion of TEXs in a mouse model. Dimethyl amiloride was proved to inhibit exosome release by several studies (Panigrahi et al., 2018; Liu et al., 2020; Peak et al., 2020). As an inhibitor of H^+^/Na^+^ and Na^+^/Ca^2+^ channels, dimethyl amiloride was considered to prevent the establishment of the calcium gradient necessary for exosome release (Savina et al., 2003; Peak et al., 2020). Therefore, dimethyl amiloride is expected to be a modulator of MDSCs. Besides, recently exosomal miRNAs effects on MDSC expansion and immunosuppression have been focused, which will be discussed in detail in the next sections.

### MiRNAs With Regulatory Effects on MDSCs

MiRNAs are a kind of endogenous non-coding small molecular RNAs that play a crucial role in the biological processes of cells, and the abnormality of their expression is a characteristic shared by many tumors (Peng and Croce, 2016). Recent studies have revealed that miRNA regulated the differentiation and expansion of MDSCs through different signaling pathways. MiR-155 and miR-21 are the two most highly expressed miRNAs in MDSC proliferation and differentiation. TGF-β promotes the expansion of MDSC by increasing miR-155 and miR-21 expression; meanwhile, miR-155 and miR-21 could exert synergistic effects on MDSC expansion, and the mechanism is STAT3 activation resulting from targeting SHIP-1 and PTEN (Li et al., 2014). The GM-CSF in the TME induces miR-200c expression, which in turn promotes the immunosuppressive effects of MDSCs. The induction of MDSCs by miR-200c is dependent on the activation of STAT3 and PI3K/Akt by targeting PTEN/friend of Gata 2 (FOG2) (Mei et al., 2015). In B lymphoma tumor-bearing mice, miR-30a promoted the expansion and immunosuppressive capacity of MDSCs through two pathways: upregulated ARG-1 expression and downregulated SOCS3 to activate STAT3 signaling (Xu et al., 2017). The PEG2 from breast cancer cells improved miR-10a expression by activating PKA signaling, and miR-10a could stimulate the amplification and activation of MDSCs through the activation of AMPK signaling (Rong et al., 2016). MiR-494 induced by TGF-β 1 in the TME increases the activity of the Akt pathway by downregulating PTEN, that is, regulating the expansion of MDSCs in tumor tissue through PTEN/Akt (Liu et al., 2012). Interestingly, in addition to upregulation, downregulation of some miRNAs could promote the function of MDSCs. Tumor-related factors promote the immunosuppression of MDSCs *in vivo* by downregulating miR-17-5p and miR-20a expression, and MDSCs transfected with miR-17-5p or miR-20a have a decreased capacity to specifically inhibit CD4^+^ and CD8^+^ T cells (Zhang et al., 2011).

Additionally, miRNAs conveyed by TEXs have also been suggested to affect the cell biology of MDSCs. After analyzing the miRNA expression profiles in these TEXs from glioma, Guo et al. (2019) found that miR-10a and miR-21 played a major role on MDSCs immunosuppression by targeting RAR-related orphan receptor alpha (RORA) and phosphatase and tensin homolog (PTEN). Similarly, exosomal miR-29a and mir-92a were transferred by TEXs to MDSCs in a mouse glioma cell model, and their transfection promoted the expansion of MDSCs by targeting high-mobility group box transcription factor 1 (HBP1) and protein kinase cAMP-dependent type I regulatory subunit alpha (Prkar1a), respectively (Guo et al., 2019). An overexpression of miR-107 was also observed in gastric cancer cells, mainly accumulated in their discharged exosomes. By TEXs, miR-107was delivered into host cell MDSCs to inhibit DICER1 and PTEN gene expression, which in turn expanded MDSCs and elevated ARG-1 expression to promote tumor escape and development (Ren et al., 2019).

### Expression and Function of LncRNAs in MDSCs

In addition to miRNAs, long non-coding (Lnc) RNAs are momentous cancer-related elements, and recent studies have shown that they were essential for the immunosuppressive function of MDSCs. A high expression of Hox antisense intergenic RNA (HOTAIR) was found in HCC, accompanied by differential expression of CCL2, and HOTAIR promoted the secretion of CCL2. Increased levels of MDSCs were also found in cell co-cultures *in vitro*, and HOTAIR was speculated to regulate CCL2 expression to induce the recruitment of MDSCs into the TME (Fujisaka et al., 2018). Tian et al. (2018) have found that the runt-related transcription factor-1 overlapping RNA (RUNXOR) was highly expressed in MDSCs isolated from tissues of patients with lung cancer. Further studies have shown that a decreased RUNXOR expression in MDSCs could lead to attenuation of their immunosuppression (Tian et al., 2018). Lnc-chop interacts with the inhibitory proteins of chop and C/EBP β to promote the activation of C/EBP β, and it promotes the immunosuppression of MDSCs in the TME by upregulating the level of ARG-1 and increasing NOX2 and COX2 expression (Gao et al., 2018). Pvt1 is an intergenic LncRNA with a high expression in multiple types of human cancers. The knockdown of LncRNA pvt1 inhibits the immunosuppression of PMN-MDSCs by decreasing ROS and ARG-1 activity *in vitro*. Further studies have found that hypoxic conditions and HIF-1α expression increased the production of Pvt1 in PMN MDSCs *in vitro* (Zheng et al., 2019). Interestingly, in addition to upregulation, downregulation of LncRNA promotes the immunosuppression of MDSCs. MALAT1 LncRNA is considered to play a significant role in tumor initiation and progression. Zhou et al. (2018) have found decreased MALAT1 expression levels in patients with lung cancer compared with healthy individuals, but MDSCs expanded and accompanied by ARG-1 level increased, demonstrating that MALAT1 negatively regulated MDSCs.

### ER Stress

The ER maintains homeostasis under normal physiological conditions by handling the folding of the secretory or transmembrane proteins. The unfavorable circumstances such as hypoxia, oxidative stress, and increased extracellular acidity, in the TME could impair the normal function of the ER and disrupt the loading and distribution of newly synthesized proteins, consequently inducing ER stress. A recent study has shown that ER stress induced apoptosis in MDSCs by upregulating TRAIL-R. This stress response promoted the further expansion of MDSCs, although it shortened their lifespan (Condamine et al., 2014). Thapsigargin, a class of highly oxidized sesquiterpene lactones isolated from the Mediterranean plant *Thapsia garganica*, has been recognized as an ER stressor because of its irreversible inhibition of the sarcoplasmic/ER Ca^2+^-ATPase pump which pump Ca^2+^ ions from the cytoplasm into ER (Jaskulska et al., 2020). Thapsigargin inhibited Ca^2+^ transport from the cytosol to ER, declining Ca^2+^ concentration in the ER to lead ER dysfunction and eventually trigger ER stress. Lee et al. (2014) have demonstrated that Thapsigargin induced persistent ER stress, which enhanced tumor-infiltrating MDSCs generation and their immunosuppression by upregulating ARG-1, iNOS, and NOX2. Further studies have found that blocking ER stress effect response by using 4-phenyl butyric acid alleviated the expansion of MDSCs in TME and tumor growth (Lee et al., 2014). Chop is a transcription factor that plays a momentous role in ER stress-induced MDSCs. Immunosuppression of tumor infiltrating chop-deficient MDSCs is attenuated, which not only failed to suppress T cells but instead induced the antitumor function of T cells. The decreased immunosuppressive function of chop-deficient MDSCs was mainly mediated by pSTAT3 downregulation, reduced IL-6 secretion, and inhibition of the C/EBP β signaling pathway (Thevenot et al., 2014). Unfolded protein response (UPR) is an adaptive response of cancer cells and tumor associated myeloid cells to cope with ER stress, restoring ER proteostasis. Recently, the regulation of UPR on MDSCs has also been paid attention by researchers. Mohamed et al. (2020) showed that tumor infiltrating MDSCs elevated pancreatic ER kinase-like ER kinase (PERK) activity, while PERK deletion converted MDSCs into cells activating CD8^+^ T cell antitumor immunity. This study suggests that relieving the UPR may reprogram the MDSCs function in TME.

### Driving Effects of Energy Metabolism on MDSCs

In recent years, with the advancement of research in the field of immunometabolism, metabolic regulation has become a hot spot in the field of immunotherapy. The level of metabolism is tightly bound to the state of cells. Previous reports have indicated that different energy metabolism pathways could produce an effect on the differentiation and biological characteristics of MDSCs in the TME. Glucose and fatty acid (FA) metabolism play a crucial role in MDSC differentiation and its suppressive effects, underscoring the potential of MDSCs as targets for immune-metabolic regulation.

Cancer cells undergo metabolic reprogramming to adapt to the TME and provide energy for their rapid proliferation. Tumor cells still tend to produce energy in the glycolysis pathway under aerobic conditions, and approximately 95% of ATP is obtained through this pathway. This phenomenon is called aerobic glycolysis (also known as the Warburg effect), which penetrates the TME and produces an effect on immune cells (Sica and Strauss, 2017). The Warburg effect is present in MDSCs during their maturation, mainly related to a high rate of glucose and glutamine uptake (Goffaux et al., 2017). Cancer cell glycolysis preferentially converts accumulated pyruvate to lactate, which could induce HIF-1α and promote MDSC generation (Chiarugi et al., 2012). Husain et al. (2013) have demonstrated that this process was supported by lactate dehydrogenase isoform A, silencing of which reduced the levels of MDSCs in a pancreatic cancer mouse model. Moreover, as a part of overall metabolism, mTOR-mediated induction of HIF-1α is necessary for glycolysis activation (Liu et al., 2014). In addition to glycolysis, lipid metabolism pathways provide energy for ATP production. Tumor-infiltrating MDSCs have been found in different murine tumor models to have increased FA uptake and activation and activated FAO, and employing FAO inhibitors could block immunosuppressive pathways and functions in MDSCs. Therefore, targeting FAO may become an effective strategy to restrict MDSCs (Hossain et al., 2015).

### MDSCs as a Therapeutic Target for Tumor Treatment

**FIGURE 2:**
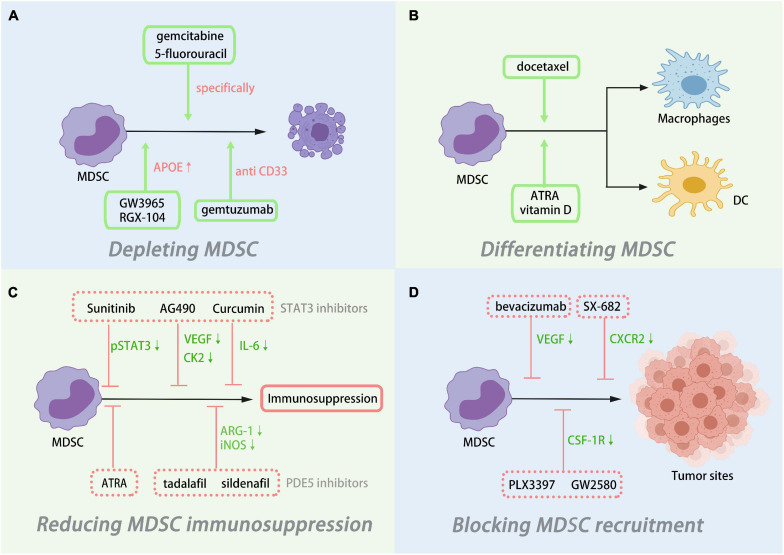
Myeloid-derived suppressor cells (MDSCs) as therapeutic targets in tumor. MDSC modulation could be achieved by **(A)** depleting MDSC, **(B)** differentiating MDSC, **(C)** reducing MDSC immunosuppression, and **(D)** blocking MDSC recruitment.

As discussed above, MDSCs are on the higher levels in various cancers compared with normal controls, such as colorectal cancer, pancreatic cancer, and so on (Markowitz et al., 2015; Limagne et al., 2016; Goldmann et al., 2017; Cha and Koo, 2020). MDSCs have been considered one of the major obstacles in cancer treatment because of their immunosuppression and non-immunologic functions. Therapeutic approaches targeting MDSCs are thriving and mainly include eliminating MDSCs, promoting MDSCs differentiation to a mature myeloid cell phenotype, attenuating the immunosuppressive function of MDSCs, as well as blocking MDSC recruitment to tumor sites (Figure 2).

Elimination of MDSCs is the most straightforward strategy for targeting MDSCs therapy. Earlier studies have shown that both gemcitabine and 5-fluorouracil specifically reduce MDSCs (Strauss et al., 2007; Vincent et al., 2010). However, a recent clinical study in pancreatic patients found that though gemcitabine in combination with omega 3 significantly reduced MDSC levels in patients, gemcitabine alone was not effective (Hou et al., 2020). Liver X receptor (LXR) induced MDSC apoptosis by activating the LXR/apolipoprotein E (APOE) axis (Tavazoie et al., 2018). Liang and Shen (2020) proved that LXR agonists GW3965 and RGX-104 enhanced antitumor immune responses and improved radiosensitive effects of non-small cell lung cancer (NSCLC) by reducing the level of tumor-infiltrating MDSCs induced by radiotherapy. CD33 is highly expressed on MDSCs in humans, Fultang et al. (2019) found that the combination of the anti-CD33 monoclonal antibody gemtuzumab and the immunotoxin ozogamicin nicely eliminated CD33^+^ MDSC, providing a novel strategy in targeting MDSCs treatment.

Additionally, promoting the differentiation of MDSCs into mature myeloid cells is another effective targeting MDSCs therapy. All-trans retinoic acid (ATRA) produced via vitamin A metabolism was the first therapeutic compound used to target MDSCs. ATRA upregulated glutathione (GSH) expression, which suppressed ROS levels in MDSCs, thereby promoting their differentiation. In addition, ATRA decreased the expression of immunosuppressive genes mediated by MDSCs, including PD-L1, IL-10, and IDO, thereby downregulating their immunosuppressive effects (Tobin et al., 2018). Fleet et al. (2020) found that MDSCs in TME had higher vitamin D receptor levels, and active vitamin D3 may also promote the differentiation of MDSCs. In the mouse model of breast cancer, docetaxel administration polarized mouse spleen MDSCs to M1-like macrophages with anti-tumor activity (Kodumudi et al., 2010).

Reducing the immunosuppressive function of MDSCs can also reduce its tumor promoting effect. STAT3 signaling pathway is an indispensable loop in the tumor development promoted by MDSCs. Sunitinib, AG490, and Curcumin attenuated the immunosuppressive function of MDSCs mainly through negative regulation of STAT3 (Ko et al., 2010; Liu et al., 2018; Salminen et al., 2018). The IL-6 in the TME induces MDSCs to mediate tumor immune escape through different pathways. Liu et al. (2016) have demonstrated that curcumin downregulated the levels of IL-6 in tumor tissues to impair MDSC function, thus significantly inhibiting tumor growth in Lewis lung carcinoma tumor models.

Furthermore, there are studies focused on blocking MDSC migration to tumor sites. VEGF favors the accumulation of MDSCs into tumor tissue and contributes to tumor development by promoting tumor angiogenesis. Bevacizumab was used for anti-VEGF treatment in patients with NSCLC. Compared with that in the non-bevacizumab regimen, the level of PMN-MDSCs was significantly decreased (Koinis et al., 2016). CSF-1 recruits MDSCs with the ability to support tumor immune escape by binding to CSF-1R expressed by MDSCs, and studies demonstrated that selective inhibitors PLX3397 and GW2580 could block their signaling by targeting CSF-1R (Priceman et al., 2010; Mok et al., 2014). Targeting the specific chemokine receptor CXCR2 on MDSCs also prevented MDSCs recruitment to tumor tissues, treatment with the CXCR2 inhibitor SX-682 reduced MDSCs migration to TME and improved the efficacy of anti-PD1 therapy (Highfill et al., 2014).

## Conclusion

As research on MDSCs has progressed, the expansion and activation of MDSCs appear to be a universal feature in malignant tumors, highlighting the importance of understanding their biological functions in the TME. In this review, the facilitative roles of TME on MDSC recruitment expansion and immunosuppression were highlighted. The suppression of their pro-tumorigenic effects by changing the favorable conditions in the TME for MDSC development may provide a new direction for MDSC-targeted antitumor therapy. Given the multiple tumor-promoting effects of MDSCs, their targeting becomes an attractive option. But there are still many problems to be solved for the clinical application of MDSC-targeted therapy in cancer. MDSCs have multiple subpopulations and exhibit high heterogeneity in different tumors. Therefore, more in-depth studies are needed to find specific markers under different tumor contexts so as to understand MDSCs more accurately. Emerging bulk or single-cell genomics analyses are perhaps providing a direction for the identification of MDSCs, but more robust experimental validation is needed. Second, MDSCs have a short- lifespan in tissues, so it is difficult to alleviate the tumor by reversing the pathological activation of tissue MDSCs. Therefore, effective therapies could aim to block MDSCs differentiation in the bone marrow, inhibit their migration to the affected tissues, or by manipulating the tissue microenvironment. More importantly, the TME is so complex that multiple immune cells and cytokines derived from multiple pathways constitute a complex network. Treatments that targeting MDSCs alone are difficult to achieve perfect therapeutic outcomes. So, it is necessary to consider combining with other treatment schemes to achieve the best therapeutic effect. For instance, the combination of LXR agonists and radiotherapy has shown a positive therapeutic effect in NSCLC, which is a promising prospect (Liang and Shen, 2020). Future studies are required to further unravel the intricacies of MDSC tumor-promoting pathways and provide a more reliable basis for targeting MDSCs alone and in combination with immunotherapy regimens.

## Author Contributions

YL wrote the manuscript. YL, HH, RJ, and JZ participated in the manuscript content collation. HY and RZ contributed to revisions of the manuscript. All authors contributed to the article and approved the submitted version.

## Conflict of Interest

The authors declare that the research was conducted in the absence of any commercial or financial relationships that could be construed as a potential conflict of interest.

## Publisher’s Note

All claims expressed in this article are solely those of the authors and do not necessarily represent those of their affiliated organizations, or those of the publisher, the editors and the reviewers. Any product that may be evaluated in this article, or claim that may be made by its manufacturer, is not guaranteed or endorsed by the publisher.
